# Death from Ulceration of the Appendix Vermiformis

**Published:** 1837

**Authors:** Wolcott Richards

**Affiliations:** Cincinnati


### Art. IV.—Death from Ulceration of the Appendix Vermiformis.

*A Case of Death from Ulceration of the Appendix Vermiformis.* By Wolcott Richards, M. D., of Cincinnati.

August 25th, 1837, called to see Mr. M——, who, I am informed, was seized the day before with chill, followed by fever. He is about 35 years of age. His general health good, with the exception of a slight dry cough, with which he has been afflicted for nine or ten months; unattended, however, by pain, fever, or any other symptom of pulmonary disease. He has head-ache, furred tongue, pain in his back and limbs, hot and dry skin, pulse about 90, and soft. Gave an emetico-cathartic, which operated freely. He vomited considerable bile; evacuations from the bowels dark-green and offensive.

26th, A. M., symptoms much the same; passed a restless night. Ordered 20 grains calomel, to be followed by calcined magnesia. Bowels freely operated upon. Evacuations watery, but of a better color.

37th, A. M., head-ache nearly gone; pain in the back and limbs removed; tongue still furred and moist; skin for the most part dry, though he says it has been moist at intervals; pulse about the same in frequency, soft. Gave calomel 2 grains, pulv. antimonials 5 grains, nit. potasi 2 grains, mixed, every four hours. Continued this treatment till the third of September, with slight modifications, during which time his symptoms became daily more favorable. On the morning of the third, found him free from fever; skin very moist; tongue cleaning; pulse about 80, but quite small; complained of great debility. Ordered chicken broth—medicines to be discontinued.

Sept. 3d, P. M., not so well; face flushed, tongue dry, pulse quick; upon inquiry, found he had taken half a pint of claret, and eaten some chicken. Ordered calcined magnesia.

4th, A. M., quite comfortable, excepting cough, which has continued in a slight degree through his sickness. Ordered an emulsion of extract of liquorice and gum arabic, with one-twelfth of a grain of tart. antimony every two hours; anodyne at night.

5th, A. M., quite comfortable; cough abated; found him seated at table, enjoying a breakfast of beef-steak, toast and coffee; says he feels well, excepting extreme weakness; free perspiration; tongue clean; pulse about 90, and feeble. Desired him not to eat beef-steak, but to confine himself to broth, rice, toast, &c.

Sept. 6th, 10 A. M., called to see my patient, expecting to find him sitting up; but to my astonishment found him presenting the appearance of a man in the collapse of Asiatic cholera. He was in a most profuse sweat, which had wet his pillow and mattress through a doubled sheet; surface cold as marble; pulse 140, and very feeble; considerable difficulty of breathing: extreme tenderness of whole abdomen; pain and slight distension of the hypogastric region. He said these symptoms suddenly supervened the evening before, without any assignable cause, and had continued more and more aggravated ever since. Sent for me in the evening, but could not find me. How to account for this entire change in the condition of the patient within 24 hours, I was quite at a loss. He said he had eaten nothing since the breakfast before mentioned; that he took no dinner, and but a cup of tea at night. I concluded there must either be retention of urine or some organic lesions in the abdominal cavity. Ascertained that he had passed no urine for 18 hours. The bowels were opened freely in the evening, and again moderately this morning. The first evacuation was quite solid; the last rather thin; both of a healthy color.

At 11 o'clock, A. M., Dr. Drake visited him with me. A catheter was introduced into the bladder, but little urine was found.

Upon further inquiry of the boy who had charge of him, I ascertained that on the previous evening, just before these distressing symptoms supervened, the patient had eaten two very large apples, had sucked the juice of one or more lemons, swallowing some of the peel, and that he had drank most freely of iced water through the night. This was not deemed a sufficient explanation of his present alarming condition. Still it seemed important to evacuate the bowels. Gave an injection of castor oil and oil turpentine, and stimulated the surface of the body with oil of turpentine. The injection soon passed away, bringing a few small lumps of feculent matter.

8 P. M., medicine has not operated. Symptoms rather aggravated. Ordered the medicine to be repeated.

Sept. 7th, 7 A. M., symptoms much the same. Difficulty of respiration increased; great distension and tenderness of the whole abdomen; surface cold and wet; tongue not much furred; pulse 150, and scarcely perceptible; considerable thirst; no operation from the bowels; urine passes freely, but involuntarily. Ordered five drops croton oil, and one ounce castor oil, made into an emulsion, with loaf sugar and gum arabic. From this time, the difficulty of breathing became greater and greater, the pulse more and more fluttering, till 2 P. M., when he died.

*Post Mortem Examination.*—Eighteen hours after death, the body was examined by Dr. Drake, in presence of several medical gentlemen, when it presented the following appearances: The abdomen was greatly distended, and upon pressing it slightly, a dark colored fluid issued from the nose and mouth. Upon opening the abdomen, the surface of the bowels presented a highly inflamed appearance. The upper portions of the intestines slightly red; but upon tracing the canal downward, the redness increased to a bright scarlet, and from this to a crimson and purplish hue. Portions of the small intestines ware agglutinated by coagulated lymph; omentum inflamed; bowels distended with gas; pelvis filled with fœcal matter, which, upon handling the bowels, was seen issuing from about the ileo-cæcal portion of large intestines. At length a large ragged ulcer in the appendix vermiformis was discovered. It had perforated all the coats. Through this ulcer the fœcal matter had escaped, giving rise to the intense inflammation of the peritoneal coat of the bowels. The mucous tissue of the bowels, excepting at the point of ulceration, was of healthy aspect.

Upon examination of the stomach, the upper or cardice portion was found highly inflamed, with a copious effusion of coagulable lymph; spleen much enlarged, dimensions 8 1-2 by 6 inches; liver pale, and easily torn.

There are two or three remarkable features in this case, which are worthy of notice.

1st. Notwithstanding the high degree of peritoneal inflammation, and the intense inflammation of the mucus membrane of the stomach, there was no irritability of this organ throughout his illness. He did not vomit, or complain of nausea from the time the emetic operated at the commencement of his disease, until a few moments before death, when he evacuated a mouthful of dark fluid, such as described above, and which I supposed to be the liquorice emulsion which he had taken.

2d. Though the ulceration of the bowels must have been of some standing, he did not complain of pain in that region during his illness, neither was there the slightest tenderness on pressure till the time when the alarming symptoms supervened. At this period, supposing there must be lesion of some portion of the bowels, I inquired if he had been subject to pain there, in times past? He replied that he had been very subject to it. I then inquired if he had suffered from diarrhœa, or any derangement of the bowels? and he said never—that habit in that respect had been uniformly regular.

September 1837.

